# MAGE-A3 Is a Clinically Relevant Target in Undifferentiated Pleomorphic Sarcoma/Myxofibrosarcoma

**DOI:** 10.3390/cancers11050677

**Published:** 2019-05-15

**Authors:** Anthony P. Conley, Wei-Lien Wang, John A. Livingston, Vinod Ravi, Jen-Wei Tsai, Ali Ali, Davis R. Ingram, Caitlin D. Lowery, Christina L. Roland, Neeta Somaiah, Patrick Hwu, Cassian Yee, Vivek Subbiah, Andrew Futreal, Alexander J. Lazar, Shreyaskumar Patel, Jason Roszik

**Affiliations:** 1 Department of Sarcoma Medical Oncology, The University of Texas MD Anderson Cancer Center, 1515 Holcombe Blvd., Houston, TX 77030, USA; JALivingston@mdanderson.org (J.A.L.); vravi@mdanderson.org (V.R.); NSomaiah@mdanderson.org (N.S.); spatel@mdanderson.org (S.P.); 2 Department of Pathology, The University of Texas MD Anderson Cancer Center, 1515 Holcombe Blvd., Houston, TX 77030, USA; wlwang@mdanderson.org (W.-L.W.); jwtsai0317@gmail.com (J.-W.T.); ali.ali@bcm.edu (A.A.); DRIngram@mdanderson.org (D.R.I.); caitlin.d.may@gmail.com (C.D.L.); alazar@mdanderson.org (A.J.L.); 3 Department of Surgical Oncology, The University of Texas MD Anderson Cancer Center, 1515 Holcombe Blvd., Houston, TX 77030, USA; CLRoland@mdanderson.org; 4 Department of Melanoma Medical Oncology, The University of Texas MD Anderson Cancer Center, 1515 Holcombe Blvd., Houston, TX 77030, USA; phwu@mdanderson.org (P.H.); CYee@mdanderson.org (C.Y.); 5 Department of Investigational Cancer Therapeutics, The University of Texas MD Anderson Cancer Center, 1515 Holcombe Blvd., Houston, TX 77030, USA; VSubbiah@mdanderson.org; 6 Department of Genomic Medicine, The University of Texas MD Anderson Cancer Center, 1515 Holcombe Blvd., Houston, TX 77030, USA; AFutreal@mdanderson.org

**Keywords:** MAGEA3, MAGE family member A3, cancer testis antigen, immunotherapy, adoptive T cell therapy, sarcoma, pleomorphic sarcoma, undifferentiated pleomorphic sarcoma, myxofibrosarcoma, malignant fibrous histiocytoma, tissue microarray

## Abstract

Melanoma-associated antigen 3 (MAGE-A3) expression is generally restricted to the placenta and germline cells of the testis, but it may also be expressed in sarcoma and other cancers and is associated with poor prognosis. Immunotherapy approaches targeting MAGE-A3 in other cancers have shown mixed results in the clinic, however, use of cancer testis antigens such as MAGE-A3 may have therapeutic value in the treatment of soft tissue sarcomas. Based on the recent success of anti-programmed death-1 (PD-1) therapy in undifferentiated pleomorphic sarcoma, we hypothesize that MAGE-A3-based immunotherapies may also provide benefits in this sarcoma type. We analyzed MAGE-A3 expression of sarcoma subtypes available in the Cancer Genome Atlas and Cancer Cell Line Encyclopedia and show that undifferentiated pleomorphic sarcoma/myxofibrosarcoma (UPS/MFS) expresses this potential target gene. We have identified high protein expression by tissue microarray of 106 UPS cores. We also found that high MAGE-A3 mRNA and protein expression is associated with worse overall survival in UPS/MFS. Furthermore, our results show no human leukocyte antigen (HLA) expression loss and relatively high lymphocyte infiltration by lymphocyte specific protein tyrosine kinase (LCK) marker expression. Based on these results, we propose targeting MAGE-A3 in UPS/MFS by immunotherapy techniques.

## 1. Introduction

Melanoma-associated antigen 3 (MAGE-A3) is located on chromosome Xq28 and encodes the MAGE-A3 protein. As a member of the MAGE gene family, the MAGE-A3 protein is one of several MAGE proteins capable of binding with and enhancing E3 Really Interesting New Gene (RING) ubiquitin ligase activity [1]. Similarly to other cancer-testis antigens, expression of MAGE-A3 is usually restricted to the placenta and germline cells of the testis [2], but frequently overexpressed in multiple tumor types including melanoma [3] and lung cancer [4]. In addition, presence of this antigen has been associated with worse prognosis in colorectal cancer [5], gastric cancer [6], non-small cell lung cancer [7], cutaneous squamous cell carcinoma [8] and diffuse large B-cell lymphoma [9].

Immunotherapies targeting MAGE-A3 have shown both positive and negative results in the treatment of various cancers. An HLA-DPB1*0401-restricted MAGE-A3-specific CD4+ T cell-based adoptive cell therapy was noted to be safe, and objective responses were observed in metastatic cervical cancer, esophageal cancer, urothelial cancer, and in a patient with osteosarcoma [10]. Unfortunately, two separate clinical trials involving HLA-A-restricted MAGE-A3-specific CD8+ T cell-based cellular therapies were limited by serious toxicities due to unexpected cross-reactivity of the selected epitopes to unrelated proteins despite rigorous pre-clinical assessment for specificity of the desired target. Neurological toxicities were observed when administering anti-MAGE-A3 T-cell receptor (TCR)-engineered T cells recognizing epitopes in MAGE-A3, -A9 and -A12, potentially due to expression of MAGE-A12 in the human brain [11]. Expression of MAGE-A12 in the brain was not known prior to their study. Despite this unexpected toxicity, five of nine patient developed objective responses including one patient with synovial sarcoma. Another study involving a T cell receptor (TCR) for an HLA-A*01 restricted MAGE-A3 peptide (EVDPIGHLY) resulted in two fatal cardiac toxicities thought to be associated with a peptide sequence from a muscle protein, titin (ESDPIVAQY) [12,13]. Furthermore, adjuvant treatment with a recombinant MAGE-A3 protein and AS15 immunostimulant (a liposomal formulation involving a combination of monophosphoryl lipid A and a TLR-9 agonist) did not increase disease-free survival in surgically resected non-small cell lung cancer [14] or in the treatment of surgically treated melanoma patients with stage IIIB or IIIC disease [15].

MAGE-A3 is expressed in sarcomas [16], however, the use of this antigen as a therapeutic target for sarcomas has been very limited. Another cancer-testis antigen, New York esophageal squamous cell carcinoma 1 (NY-ESO-1), as an effective immunotherapy target in synovial cell sarcoma, has demonstrated objective responses in excess of 50% of patients treated on two separate trials evaluating synovial cell sarcomas expressing NY-ESO-1 with NY-ESO-1 specific autologous TCR-transduced T cells [17,18]. The SARC028 phase II study of single-agent pembrolizumab (anti-programmed death-1) antibody in multiple sarcoma types showed an objective response rate (ORR) of 40% in UPS [19,20]. A separate trial involving nivolumab alone and in combination with ipilimumab demonstrated responses in undifferentiated pleomorphic sarcoma (UPS) [21]. The success of these immune checkpoint inhibitor trials in UPS indicates that other immunotherapeutic options may also provide benefits to UPS patients. As the results of these trials show, immunotherapy response rates greatly vary in sarcoma subtypes.

The aim of the current study was to identify which sarcoma types express the MAGE-A3 antigen using publicly available tumor and cancer cell line sequencing data and confirm protein expression by tissue microarray (TMA) staining. We have also analyzed normal tissue expressions to show that expression of this gene is restricted to testis. As loss of antigen presentation is a key limiting factor of immunotherapies, we have analyzed HLA expression and availability of lymphocytes in sarcoma subtypes. 

## 2. Results

### 2.1. MAGE-A3 Is Expressed in Multiple Cancers and a Limited Number of Normal Tissues

**Figure 1:**
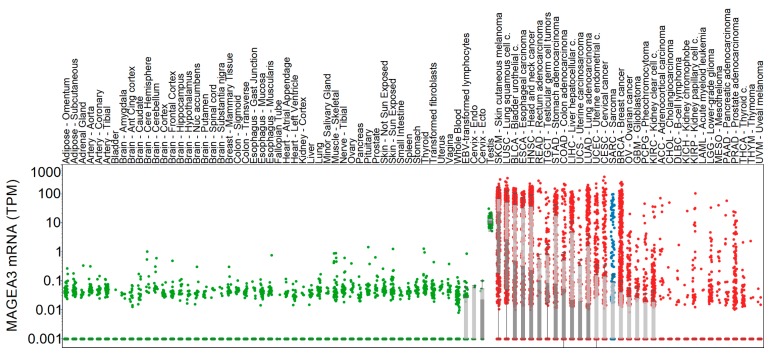
Melanoma-associated antigen 3 (MAGEA3) expression in normal and tumor tissue samples. Normal tissue (green) and tumor (red) expression of MAGEA3 is shown on the y axis, each dot representing a sample. Sarcoma samples are highlighted with blue color. Light and dark grey boxes denote the two quartiles around the median expression. Boxes are not shown where MAGEA3 median expression is zero.

MAGE-A3 mRNA expression was available from thirty-three cancers accessed from The Cancer Genome Atlas Program (TCGA). MAGE-A3 expression from all represented cancer genomes (*n* = 33) was compared with MAGE-A3 mRNA expression from normal tissues in the Genotype-Tissue Expression (GTEx) (*n* = 53, total of 8555 samples) database. Figure 1 illustrates the mRNA expression of MAGE-A3 across these normal tissues and cancer datasets. Among normal tissues, MAGE-A3 mRNA expression is highest within the testes (median expression is 11.48 transcripts per million (TPM)). By comparison, expression in ovaries was significantly lower (*p* < 0.001, median expression is 0 TPM, highest expression is 0.397 TPM). Several outliers with elevated MAGE-A3 mRNA expression are noted in samples representing skeletal muscle. Among the represented malignancies, cutaneous melanoma and squamous cell carcinoma of the lung exhibit the highest expression of MAGE-A3. As a group, sarcomas have variable expression of MAGE-A3 and exhibited higher expression compared to seventeen other cancers.

### 2.2. MAGE-A3 mRNA Is Over-Expressed in UPS/MFS

**Figure 2:**
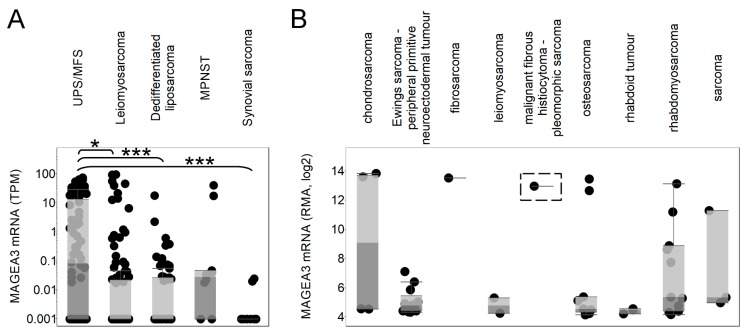
Melanoma-associated antigen 3 (MAGEA3) expression in sarcoma subtypes. Tumor tissue expression of MAGEA3 is shown for undifferentiated pleomorphic sarcoma/myxofibrosarcoma (UPS/MFS), leiomyosarcoma, dedifferentiated liposarcoma, malignant peripheral nerve sheath tumor (MPNST), and synovial sarcoma (**A**). MAGEA3 sarcoma cell line expressions are compared for chondrosarcoma, Ewings sarcoma, fibrosarcoma, leiomyosarcoma, pleomorphic sarcoma (highlighted with dashed box), osteosarcoma, rhabdoid tumor, rhabdomyosarcoma, and other unspecified sarcoma (**B**). Each dot represents a tumor sample or cell line. * *p* < 0.05, *** *p* < 0.001.

MAGE-A3 mRNA expression was available in the TCGA and the Cancer Cell Line Encyclopedia (CCLE) for 106 leiomyosarcoma tumors (LMS), 76 undifferentiated pleomorphic sarcoma/myxofibrosarcomas (UPS/MFS), 58 dedifferentiated liposarcomas (DDLPS), 10 synovial sarcomas, 10 malignant peripheral nerve sheath tumors (MPNST), and 46 various sarcoma cell lines. We found that MAGEA3 was highly expressed in UPS/MFS; the expression in these samples was significantly higher than in LMS (*p* < 0.05), DDLPS (*p* < 0.001), and synovial sarcoma (*p* < 0.001). Figure 2 illustrates the differential expression of MAGEA3 among the selected soft tissue sarcomas. By comparison, the CCLE showed variable expression of MAGEA3 among a diverse number of sarcoma cell lines. Of note, only one cell line (GCT) representing pleomorphic sarcomas was contained in the CCLE database, and that cell line shows high MAGEA3 expression.

### 2.3. High MAGEA3 Protein Expression is Noted in UPS and Correlates with Overall Survival

**Figure 3:**
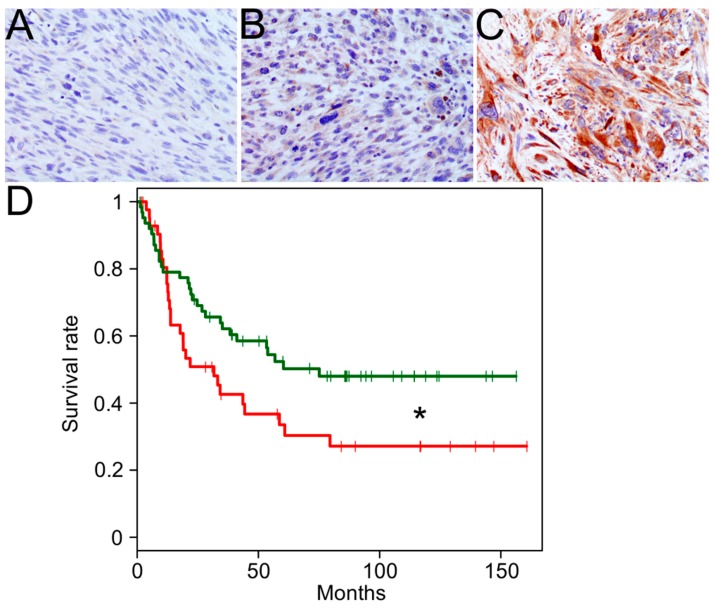
Melanoma-associated antigen 3 (MAGEA3) Expression by immunohistochemistry in UPS. Representative staining from a 106-core tissue microarray is shown for no MAGEA3 expression (**A**), weak MAGEA3 expression (**B**), and strong MAGEA3 expression (**C**). A significant association between MAGEA3 protein expression and overall survival is denoted by * (red: ≥90 extent %, *n* = 43; green: <90 extent %, *n* = 63; *p* < 0.05) (**D**).

MAGEA3 immunohistochemical study was evaluable in 106 cores. Forty-three cases (41%) exhibited high expression of MAGEA3 while 63 cases (59%) exhibited low (*n* = 59) to no (*n* = 11) expression of MAGEA3 (see Figure 3A–C). All cases with at least moderate intensity staining and the majority of cases with low intensity staining had labeling in >50% of tumor cells. High expression was more likely to seen in recurrences (56%, *n* = 24/43), than primary tumors (30%, *n* = 13/44) or metastases (31%, *n* = 6/19) (*p* = 0.03). Utilizing our UPS TMA, we show that MAGEA3 protein expression (≥90 extent %, *n* = 43 vs. <90%, *n* = 63) is associated with an adverse survival (*p* < 0.05; Figure 3D).

### 2.4. Sufficient HLA Expression and Lymphocyte Infiltration in UPS/MFS

**Figure 4:**
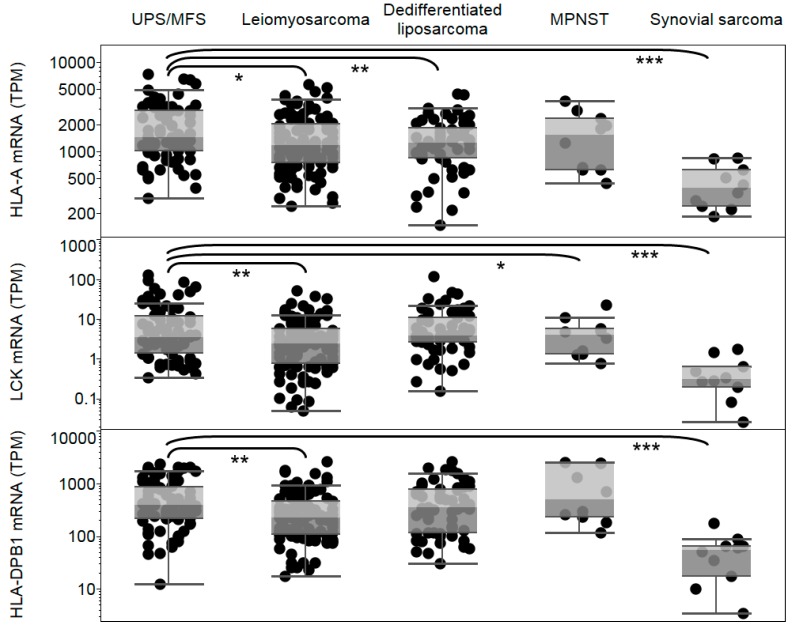
Human Leukocyte Antigen-A (HLA-A), Human Leukocyte Antigen-DPB1 (HLA-DPB1), and lymphocyte specific protein tyrosine kinase (LCK) expression in sarcoma subtypes. Expression of HLA-A, HLA-DPB1, and LCK are shown for undifferentiated pleomorphic sarcoma (UPS/MFS), leiomyosarcoma, dedifferentiated liposarcoma, malignant peripheral nerve sheath turmo (MPNST), and synovial sarcoma subtypes. Each dot represents a tumor sample. * *p* < 0.05, ** *p* < 0.01, *** *p* < 0.001.

Expression of HLA-A, HLA-DPB1, and lymphocyte marker LCK were analyzed in TCGA sarcoma subtypes to determine whether a significant loss of antigen presentation in UPS/MFS could limit efficacy of immunotherapies targeting MAGEA3. We found that HLA-A expression was significantly higher in UPS/MFS compared to leiomyosarcoma (*p* < 0.05), dedifferentiated liposarcoma (*p* < 0.01), and synovial sarcoma (*p* < 0.001) (see Figure 4). Given the prior use of HLA-DPB1-restricted MAGE-A3-specific CD4+ T cells, we analyzed mRNA expression of HLA-DPB1 and found that expression was significantly higher in UPS/MFS compare to leiomyosarcoma (*p* < 0.01) and synovial sarcoma (*p* < 0.001). Lymphocyte infiltration was estimated through LCK expression. We observed significantly higher LCK in UPS/MSF compared to leiomyosarcoma (*p* < 0.01), MPNST (*p* < 0.05), and synovial sarcoma (*p* < 0.001) (Figure 4).

### 2.5. MAGEA3 mRNA Expression in UPS/MFS Correlates with Overall Survival

**Figure 5:**
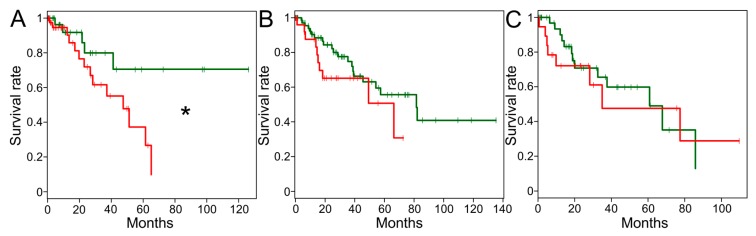
Correlation of Melanoma-associated antigen 3 (MAGEA3) expression with overall survival. Kaplan–Meier plots comparing undetectable (zero transcripts per million [TPM], green), and detectable MAGEA3 expression (greater than zero, red) are shown for undifferentiated pleomorphic sarcoma/myxofibrosarcoma (UPS/MFS) (**A**), leiomyosarcoma (**B**), and dedifferentiated liposarcoma (**C**). A significant association between MAGEA3 mRNA expression and overall survival is denoted by * (*p* < 0.05).

Utilizing the sarcoma TCGA data set, a survival analysis was performed across all histologic subtypes for which a sufficient amount of samples and survival data were available. In the UPS/MFS dataset, samples with detectable MAGEA3 mRNA expression were associated with an inferior survival compared to samples with no detectable MAGEA3 mRNA expression levels (Figure 5, *p* < 0.05, detectable: *n* = 44, median survival = 47.5 months; undetectable: *n* = 31, median survival was not reached). For no other subtype was a survival difference observed based on MAGEA3 mRNA expression levels (leiomyosarcoma: detectable *n* = 29, undetectable *n* = 75; dedifferentiated liposarcoma: detectable *n* = 18, undetectable *n* = 40).

## 3. Discussion

Although expression of cancer-testis antigens, presence of various fusion and mutated proteins, as well as immune infiltrate have been reported in soft tissue sarcomas, current immunotherapy options are limited for these patients [22]. The results of several successful immunotherapy trials using an anti-programmed death-1 (PD-1) antibody and combination anti-PD-1/anti-cytotoxic T-lymphocyte associated protein 4 (CTLA-4) show that a significant number of UPS patients experience a reduction in tumor size [21,23]. This underlies the need for identification and testing of other immunotherapy methods and targets that may be effective in UPS. Promising markers and therapeutic targets have been identified in UPS and MFS using primary cultures [24,25], and correlation of oncologic outcome and clinicopathologic variables as well as expression of various markers has been also studied [26]. In addition, a small study of 17 patients treated with neoadjuvant radiation therapy prior to resection, immune cell infiltrates and PD-L1 expression were observed to increase with radiation treatment [21]. Given the limited scope of this study, cancer-testis antigen evaluation was not performed. As many different histological and molecular sarcoma subtypes exist [27], it is challenging to predict and test which subtypes would benefit from immunotherapies.

In the last years, large amounts of next-generation sequencing data have become available from the Cancer Genome Atlas and other projects [28], including the Cancer Cell Line Encyclopedia for cancer cell lines [29]. Furthermore, normal tissue expression data for more than 8000 normal samples are accessible from the Genotype-Tissue Expression project [30]. The availability of these databases allows researchers to determine which potential therapy targets are expressed in which cancers, and analyze correlations with survival and other clinical variables [31]. Our goal was to assess the availability of MAGEA3 as a target in sarcoma subtypes that were included in the above mentioned projects, and to confirm expression of this target at the protein level.

MAGEA3 is an attractive immunotherapy target in multiple cancers, and its expression in sarcoma predicts that adoptive T cell therapies or cancer vaccines may be successfully applied in specific sarcoma subtypes that express this antigen. Our analysis of large, publicly available tumor (Cancer Genome Atlas) and cancer cell line (Cancer Cell Line Encyclopedia) databases shows that MAGEA3 is expressed in UPS/MFS. Our 106-core tissue microarray staining has shown that not only the MAGEA3 gene, but also the encoded protein is expressed in this sarcoma subtype. Analysis of MAGEA3 expression in the Genotype-Tissue Expression (GTEx) project confirmed that MAGEA3 is generally restricted to testis. Furthermore, our Kaplan–Meier analyses showing an association of detectable MAGEA3 mRNA and immunohistochemical (IHC) protein expression with shorter survival and was seen more likely in recurrences by our immunohistochemical study, supporting the relevance of this target. Other histologic subtypes showed significantly lower MAGEA3 expression compared to UPS/MFS, and this may contribute to the lack of survival association in those subtypes.

HLA-A mRNA expression and HLA-DPB1 mRNA expression were found to be significantly higher in UPS/MFS compared to other sarcomas, suggesting that efficacy of TCR-based immunotherapies targeting MAGEA3 may not be limited by loss of HLA expression. The success of the recent immune checkpoint inhibitor studies in soft tissue sarcoma [19,21] forecasts that immunotherapy for UPS is feasible, and our results also predict that lymphocytes are present in UPS/MFS tumors. Therefore we consider MAGEA3 a clinically relevant target in UPS/MFS, and envisage that a subset of patients will benefit from immunotherapies targeting this antigen. A limitation of this study is that we had only UPS and no MFS cases in our immunohistochemistry analysis. Although recognized by the WHO as distinct separate identities, we were unable to separate UPS and MFS into two distinct groups using the sarcoma TCGA. The reason for this is that UPS and MFS were found by the TCGA project to be molecularly similar with a continuum in terms of myxoid component [32].

## 4. Materials and Methods

### 4.1. RNA Sequencing Data

Gene expression and clinical data from the sarcoma TCGA were downloaded from public TCGA repositories [32]. In the TCGA, the following sarcoma histologies were represented: Leiomyosarcoma (*n* = 106 samples), undifferentiated pleomorphic sarcoma/myxofibrosarcoma (UPS/MFS) (*n* = 76), dedifferentiated liposarcoma (DDLPS) (*n* = 58), synovial sarcoma (*n* = 10), and malignant peripheral nerve sheath tumors (MPNST) (*n* = 10). Normal tissue expressions from RNA sequencing were obtained from the Genotype-Tissue Expression (GTEx) project [30]. We used the transcripts per million unit to compare gene expressions from RNA sequencing [33]. Cancer cell line expressions were obtained from the Cancer Cell Line Encyclopedia (CCLE) [29]. From the CCLE, 46 sarcoma cell lines were available for analysis. This retrospective biomarker study was reviewed and approved by the University of Texas MD Anderson Cancer Center Institutional Review Board (IRB). The use of the tissue and clinical data were covered under protocol LAB04-0890 approved on 5 November 2007.

### 4.2. Immunohistochemistry (IHC)

With institutional review board approval (LAB04-0890 protocol, approved by the MD Anderson Institutional Review Board for the acquisition of tissue and clinical data, initial approval date: 11/5/2007), a tissue microarray (TMA) was constructed from 106 formalin-fixed paraffin embedded tissue undifferentiated pleomorphic sarcoma (UPS) from 90 patients. All cases were reviewed by sarcoma pathologists (Wei-Lien Wang, Jen-Wei Tsai, Alexander J Lazar). The tissue microarray consisted of 47 primary tumors, 38 recurrences, and 21 metastases. Four-micron unstained slides were prepared from the TMA blocks and stained with anti-MAGEA3 (1:100, LS-B884, LSBio, Seattle, WA, USA). Cytoplasmic labeling was assessed for both intensity (0: none, 1: mild, 2: moderate and 3: strong) and percentage of tumor cells labeled (extent %). De-identified clinical data and IHC results are provided as a Supplementary Table S1.

### 4.3. Statistical Analyses

For comparisons of two groups we performed two-tailed Student’s *t*-tests. Differences were considered significant when *p* < 0.05.

## 5. Conclusions

Based upon our findings, MAGEA3 may be a relevant therapeutic target in UPS/MFS. In addition, its expression may be prognostic in UPS though further validation is needed.

## Supplementary Materials

The following are available online at https://www.mdpi.com/2072-6694/11/5/677/s1, Table S1: De-identified clinical data and IHC results.
